# A hybrid model combining 1D-CNN and BERT for intelligent ECG arrhythmia classification

**DOI:** 10.1038/s41598-025-28023-4

**Published:** 2025-11-21

**Authors:** Hanqing Liu

**Affiliations:** School of Computer Science and Technology, Beijing Jiaotong University, Beijing, 100044 China

**Keywords:** Cardiology, Computational biology and bioinformatics, Engineering, Mathematics and computing

## Abstract

Arrhythmia is a common cardiovascular disease, whose early diagnosis is crucial to prevent severe cardiac events. Traditional electrocardiogram (ECG) interpretation methods rely on manual analysis, which often suffers from low efficiency and limited accuracy. To address these issues, intelligent algorithms are increasingly being used for automatic arrhythmia recognition. However, many existing methods still face challenges in achieving accurate classification. In this paper, we propose a novel approach that integrates a one-dimensional convolutional neural network (1D-CNN) with Bidirectional Encoder Representations from Transformers (BERT) for arrhythmia classification. The proposed model, named ECGBert, leverages the local feature extraction capability of CNN and the global context modeling strength of BERT. The model enables the precise classification of different types of arrhythmias by performing signal preprocessing, segment encoding, and sequential feature extraction. Experimental results in the MIT-BIH Arrhythmia Database demonstrate that ECGBert significantly outperforms traditional methods and existing hybrid architectures in multiple evaluation metrics. The model effectively captures long-range dependencies between abnormal heartbeats by incorporating the Transformer mechanism. It also maintains an end-to-end learning structure without the need for hand-crafted features, offering strong generalization ability and robustness. This work provides a new methodological framework for intelligent ECG analysis and promotes the innovative application of deep learning in medical signal processing.

## Introduction

Arrhythmia refers to abnormalities in cardiac rhythm or rate caused by irregular electrical activity, often due to abnormal impulse initiation or conduction block^1^. It is prevalent in patients with chronic conditions such as hypertension, coronary artery disease, and diabetes, and may result in severe outcomes including sudden cardiac death, heart failure, or stroke. For instance, atrial fibrillation increases stroke risk by four- to five-fold, posing a major global healthcare burden.

ECG is a non-invasive technique that records cardiac electrical activity and remains a cornerstone in arrhythmia diagnosis. However, manual ECG interpretation relies heavily on expert knowledge, and is inefficient and error-prone when faced with large-scale and dynamic data. This motivates the development of automated and intelligent ECG analysis methods to enhance arrhythmia detection and support clinical decision-making.

To address this challenge, researchers have focused on developing intelligent algorithms based on ECG signals to improve diagnostic accuracy. Early studies primarily relied on traditional machine learning approaches. For example, Lyon et al.^2^ investigated the application of Support Vector Machines (SVM) for high-dimensional ECG data and achieved reasonable classification performance. Random Forest (RF) models were also widely used for preliminary arrhythmia classification tasks. However, these methods exhibited significant limitations in automatic feature extraction and generalization capabilities.

With the rapid development of deep learning, its application in ECG signal processing has become increasingly prominent. Hong et al.^3^ conducted a systematic review of intelligent ECG analysis and highlighted the advantages of deep models in noise robustness, temporal modeling, and handling data imbalance. Deep learning models have made remarkable progress in arrhythmia classification tasks. For instance, Musa et al.^4^ systematically analyzed the performance of CNN, RNN, and CRNN models in ECG classification. Rajpurkar et al.^5^ proposed a CNN-based model that achieved expert-level performance in atrial fibrillation detection. Li et al.^6^ developed a model based on BiLSTM with an attention mechanism, which significantly improved classification accuracy by capturing global features from heartbeat sequences. To address the data imbalance issue, researchers also introduced focal loss functions and Generative Adversarial Networks (GANs) to mitigate the problem of misclassification in minority classes.

Despite these advancements, significant challenges remain in large-scale clinical deployment. ESC cardiovascular statistics show that the incidence of arrhythmias such as atrial fibrillation and ventricular tachycardia, along with their associated stroke risks, continues to rise^7^. Therefore, more accurate and widely applicable detection technologies are urgently needed. Danilov and Aronow^8^ pointed out that model performance varies significantly across different devices and databases, mainly due to ECG signal heterogeneity, acquisition noise, and limited feature representation capabilities. These factors reduce classification accuracy and cross-domain robustness, thereby limiting clinical reliability. Enhancing the model’s feature extraction and recognition capabilities has become a key task for improving ECG-based intelligent analysis.

To address these challenges, we propose ECGBert, a deep learning model that integrates one-dimensional time-series signals with two-dimensional image information. The model combines 1D-CNN and BERT for multi-level feature extraction, capturing both local and global characteristics of ECG signals. Experimental results demonstrate that ECGBert outperforms traditional approaches and existing hybrid models in classification accuracy, stability, and robustness, particularly in small-sample scenarios.

Validated on multiple public datasets, ECGBert eliminates manual feature engineering by learning discriminative representations in an end-to-end manner, offering a feasible and effective solution for automated arrhythmia detection with clinical potential. This work advances the application of deep learning in medical signal processing and contributes to improving the efficiency and accuracy of healthcare services.

## Related work

ECG signals, as an important type of bioelectrical signal, play a vital role in the early screening and clinical diagnosis of arrhythmias. The core of automatic arrhythmia recognition lies in extracting effective temporal and morphological features from ECG signals and constructing accurate classification models. Early studies mainly relied on handcrafted feature extraction and traditional machine learning classifiers such as SVM, RF, and k-Nearest Neighbors (k-NN)^9,10^. These approaches typically constructed feature spaces using statistical characteristics, time-frequency analysis, or morphological parameters of the QRS complex, and then trained classifiers for pattern recognition. However, such methods heavily depend on expert knowledge and often lack generalizability to complex real-world clinical data. In cross-subject and cross-device scenarios, they often suffer from low accuracy and high misdiagnosis rates^11,12^.

With the rapid development of deep learning, the end-to-end automatic feature learning capability has significantly improved the efficiency of ECG representation and the performance of classification models. Current research mainly focuses on Convolutional Neural Networks (CNNs), Recurrent Neural Networks (RNNs), and their hybrid structures. Recently, the Transformer architecture has also been introduced into ECG sequence modeling tasks. This section categorizes deep learning-based methods into two types: single-model approaches and hybrid-model methods. Many researchers have aimed to enhance feature extraction and optimize intelligent arrhythmia recognition techniques.

### Deep models based on CNNs and RNNs

CNNs are widely used in ECG signal processing due to their strength in modeling local features. They can automatically extract critical waveform characteristics such as QRS complexes, ST segment changes, and P wave absence through local receptive fields, effectively reducing reliance on manual features.Di Paolo et al.^13^ proposed a multimodal CNN framework with an adaptive attention mechanism, where ECG signals are transformed into images using HSFC and RP to enhance feature representation. Wu et al.^14^ proposed a 12-layer 1D-CNN architecture, combined with wavelet threshold denoising, to achieve high-accuracy recognition of multiple arrhythmia types. Shah et al.^15^ introduced a Selective Kernel (SK) module with variable receptive fields, enabling multiscale feature extraction across channels and enhancing robustness to complex waveform variations. Furthermore, Maweu et al.^16^ integrated interpretability mechanisms into conventional CNNs, using overlay visualization to highlight model attention to P-QRS-T components, thereby improving clinical usability and credibility. El-Ghaish et al.^17^ further adopted a multi-scale convolution structure combined with Transformer modules for sequence modeling, which enhanced the model’s sensitivity to rhythm changes. Yildirim et al.? developed a novel DNN architecture that combines representational learning and sequence modeling for arrhythmia detection.Berrahou et al.^18^ proposed a CNN-based arrhythmia classification method incorporating RR interval and entropy rate features, achieving competitive performance on both intra- and inter-patient experiments using the MIT-BIH and INCART datasets, demonstrating strong generalization ability.

While CNNs perform well in recognizing static heartbeat features, they have limitations in capturing long-term dependencies in ECG signals. To address this issue, RNNs and their variants, such as Long Short-Term Memory (LSTM) networks and Gated Recurrent Units (GRUs), have been introduced for rhythm modeling tasks. Petmezas et al.^19^ proposed a CNN-LSTM model that combines Focal Loss to address class imbalance, achieving significant improvements in recognizing confusing arrhythmia types like AFIB and AFL. Yuen et al.^20^ used CNNs to extract local waveform features, followed by LSTMs to capture dynamic rhythmic structures between heartbeats, and further refined classification boundaries using a Multi-Layer Perceptron (MLP). Jin et al.^21^ proposed the DLA-CLSTM model, which incorporates convolutional attention mechanisms to enhance feature weighting, improving rhythm recognition accuracy while maintaining model efficiency. In comparison, CNNs are better at extracting local morphological features, while RNNs are more suitable for modeling rhythmic and temporal dependencies. Their combination balances feature representation and sequence modeling, making them effective for most small- and medium-scale ECG tasks.

### Deep models based on transformer and hybrid architectures

In recent years, the Transformer architecture has gained widespread attention for its strong global modeling ability and multi-head attention mechanism. Its application in ECG signal analysis is also emerging. The Transformer uses self-attention to capture long-range dependencies, overcoming gradient vanishing and efficiency issues faced by traditional RNNs. Liu et al.^22^ proposed an end-to-end model combining CNN and Transformer. They first extracted heartbeat features from sliding windows using CNN, then applied a Transformer to model global dependencies among local rhythms, improving the accuracy of complex rhythm recognition such as atrial fibrillation. Xia et al.^23^ introduced a lightweight Transformer into a CNN and denoising autoencoder (DAE) framework to strengthen inter-beat dependency modeling. Experiments showed that this model performed well in recognizing minority class samples. Essa et al.^24^ integrated two deep learning models using a bagging strategy on training set subsets. A meta-classifier was then used to fuse the outputs, significantly reducing false positives. Admass et al.^25^ proposed an arrhythmia classification framework based on attention-driven deep learning, meta-heuristic optimization, and a custom ARR-LO algorithm, achieving efficient classification through ECG analysis.Islam et al.^26^ proposed CAT-Net, a model that combines convolution, attention, and Transformer with the SMOTE-Tomek strategy, achieving state-of-the-art performance for single-lead ECG arrhythmia classification on the MIT-BIH and INCART datasets.Anitha et al.^27^ developed a deep Bi-CapsNet model for arrhythmia classification, integrating CNN-RNN feature extraction with a bidirectional capsule network, and achieved about 97.19% accuracy on the MIT-BIH database, outperforming several conventional deep learning models.

Although traditional machine learning methods have shown some promise, they often struggle to handle noisy ECG data and lack the ability to generalize across different patient populations and acquisition devices.To further enhance model performance and generalization, multimodal and multi-branch architectures have been widely adopted. One approach extracts features from both one-dimensional time series and two-dimensional image representations, using parallel networks to fuse multi-view information and improve discriminative power. Another approach focuses on integrating diverse deep learning modules. For instance, CNNs extract local static features, GRUs model short- and medium-term rhythm variations, and Transformers handle global temporal dependencies, forming a hierarchical representation structure.A Panigrahi et al.^28^ leveraged transfer learning and an optimization algorithm (AVOA) to fuse ECG features and achieved 96.31% classification accuracy using ensemble ML classifiers.M Aboghazalah et al.^29^ integrated chaos theory with multiple feature extraction methods and traditional classifiers like SVM and Decision Tree, achieving up to 99.8% accuracy in arrhythmia detection.

The ECGBert model proposed in this study follows this line of thought. By combining CNN and Transformer structures, it builds a multi-level feature fusion framework from three perspectives: local waveform, rhythm dependency, and global semantics. The model introduces temporal encoding and graph transformation mechanisms in ECG signal representation. It uses the BERT architecture for feature encoding and employs multimodal attention mechanisms for fusion and classification. Experimental results demonstrate that the model achieves excellent performance on multiple public ECG datasets, particularly showing strong classification capability in small-sample tests.

## Method

**Fig. 1:**
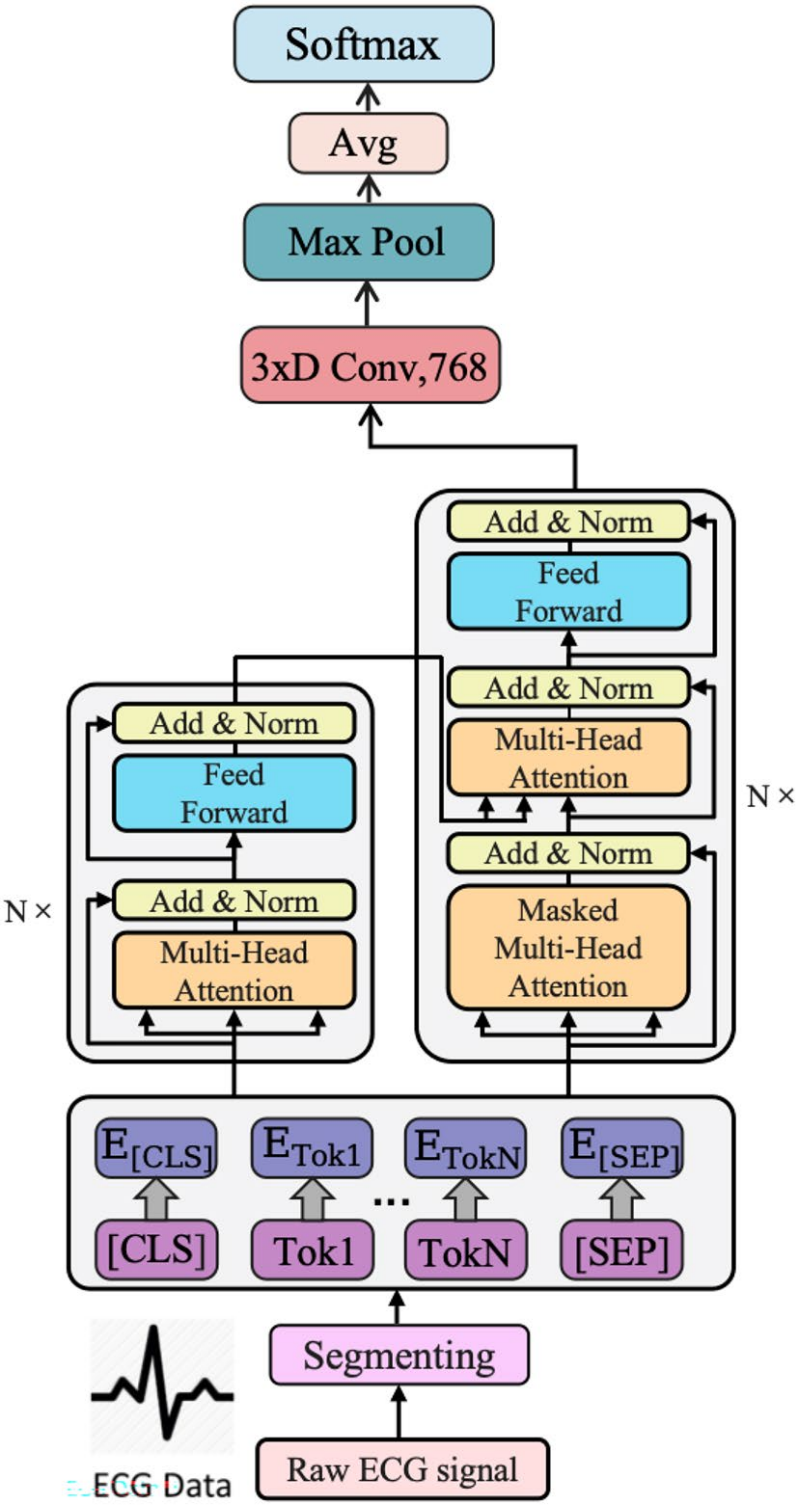
Overall architecture of the proposed ECGBert model.

The proposed **ECGBert** model consists of three key modules: a 1D-CNN local feature extraction module, a BERT global modeling module, and an optimization and loss function module for efficient arrhythmia classification. As shown in Fig. 1, the overall architecture combines the strengths of convolutional networks in capturing local structures with the ability of BERT to model long-range dependencies, enabling end-to-end learning of discriminative features directly from raw ECG signals.

### 1D-CNN module

CNNs have been widely used to process sequential and grid-structured data. In this study, we designed a feature extraction module based on 1D-CNN to capture the local temporal dependencies and morphological features within ECG signals. This module consists of multiple convolutional layers, ReLU nonlinear activation functions, pooling layers, and fully connected layers. The choice of a kernel size of 3 and a pooling window size of 2 is based on the typical morphology of ECG waveforms, ensuring the effective extraction of key features such as P waves, QRS complexes, and T waves, while also reducing dimensionality and enhancing the model’s robustness to temporal shifts.

The convolutional layers extract subtle waveform changes–such as P waves, QRS complexes, and T waves–through sliding window operations, while max pooling reduces dimensionality and enhances robustness to temporal shifts.

The model takes a 1D time series as input. The kernel size is set to 3, and multiple channels are used to extract multiscale features. The convolution operation is defined as:

1 $$\begin{aligned} y_t = \sum _{k=1}^K \omega _k x_{t-k+1} \end{aligned}$$

Where $x_{t-k+1}$ is the input signal, $\omega _k$ is the convolution kernel, and $y_t$ is the output. A ReLU activation function is then applied:

2 $$\begin{aligned} \text {ReLU}(x) = {\left\{ \begin{array}{ll} x, & x \ge 0 \\ 0, & x < 0 \end{array}\right. } \end{aligned}$$

To reduce redundancy and improve computational efficiency, a downsampling layer with a $1 \times 1$ convolution is added when the input and output channel numbers differ. Pooling operations are performed with a window size of 2, as defined by:

3 $$\begin{aligned} y_j^l = f\left( \sum _{i=1}^M \text {down}\left( x_j^{l-1}\right) + b_j^l \right) \end{aligned}$$

Here, $y_j^l$ is the feature map of the $l$-th layer, and $\text {down}(x_j^{l-1})$ represents the downsampling function. The function $f(\cdot )$ denotes the ReLU activation, and $b_j^l$ is the bias term.

A dropout layer is added to improve generalization. To match the input requirements of the BERT module, features are mapped to a fixed length via fully connected layers after convolution and pooling, enabling cross-channel fusion and consistent input formatting. This end-to-end feature extraction process eliminates the need for handcrafted features and significantly improves efficiency and accuracy.

### BERT model

To model the global semantic dependencies of ECG signals, this study introduces the Transformer architecture into the task of arrhythmia recognition and constructs a BERT-based deep learning module. We chose the BERT architecture instead of a standard Transformer encoder for two main reasons. First, BERT’s bidirectional attention mechanism allows the model to capture both past and future context, which is particularly important in ECG signals where the classification of a heartbeat depends not only on its preceding waves but also on subsequent patterns. In contrast, standard Transformer encoders often adopt a unidirectional or causal structure that may overlook such bidirectional dependencies. Second, BERT’s masked language modeling paradigm, when adapted to ECG signals, encourages the model to learn robust local-global representations by reconstructing masked signal segments.This module consists of an embedding layer, positional encoding, stacked Transformer encoders, and an attention mechanism. The input to BERT is the feature sequence extracted by a CNN, which is first passed through token embedding and positional embedding to generate a unified representation. This ensures that each time step’s feature contains both positional information and contextual semantics.

In the context of ECG sequences, we apply positional encoding to preserve the temporal order of ECG samples, ensuring that the model can distinguish between early and late features within a cardiac cycle. For example, the model can more accurately capture contextual information of key temporal segments such as P-waves, QRS complexes, and T-waves, which is helpful for detecting time-dependent abnormalities such as atrial fibrillation or premature ventricular contractions.

The core of BERT is a multi-layer stack of Transformer encoders, where each layer is composed of a multi-head self-attention mechanism and a feedforward neural network. The attention mechanism enables the model to leverage the full sequence context when encoding any given time step, thus modeling long-range dependencies. The outputs from multiple attention heads are concatenated and passed through a feedforward layer, with residual connections and layer normalization applied to stabilize gradient propagation. We employ four encoder layers, each with four attention heads. Each attention head has a dimensionality of 64, resulting in a total embedding size of 256, balancing model expressiveness and computational efficiency.

During the forward pass, the input is first projected into an embedding space through a convolutional embedding layer. After adding positional encoding, the sequence is processed by multiple Transformer layers. Within each layer, the attention mechanism dynamically adjusts attention weights based on the global characteristics of the ECG sequence, enabling the model to focus on abnormal signal segments and key cardiac moments such as R-peaks and QRS intervals. The output corresponding to the [CLS] token is used as the overall sequence representation for final classification. In addition, a lightweight convolutional layer is appended at the end of the BERT module to compress the sequence representation and adapt it to the input format required by the CNN classification head, thus achieving local refinement and feature integration.

The BERT model is capable of processing input sequences of arbitrary length. Each element in the input sequence *E* is first embedded into a 768-dimensional vector. These vectors are then sequentially processed by Transformer encoders. Through linear projections, each vector is transformed into a set of key (*K*), query (*Q*), and value (*V*) vectors, each of dimension 64, resulting in 12 attention heads. The attention mechanism is defined as:

4 $$\begin{aligned} \text {Attention}(Q, K, V) = \text {softmax} \left( \frac{QK^\top }{\sqrt{d_k}} \right) V \end{aligned}$$

where *Q*, *K* and *V* represent the query, key, and value matrices respectively, and $d_k$ is the dimension of the key vector. The dot product of *Q* and $K^\top$ computes the similarity between query and key, and the softmax function converts the similarities into a normalized weight distribution, enabling attention output computation.

Our BERT module is a self-attention-based encoder model. During initialization, it constructs a 1D convolutional layer, an embedding layer, and a list of encoder blocks. The convolutional layer projects the input sequence into the model’s representation space. The embedding layer generates positional encodings, which are added to the input representation to provide position information. The encoder block list contains multiple encoder layers, forming the core of the BERT encoder. The *K*, *Q*, and *V* matrices are passed through 12 self-attention heads, producing 64-dimensional vectors that capture the sequence-wide contextual information. The outputs of the attention heads are combined and passed through linear and feedforward layers to increase non-linearity and model depth. This process is repeated through all stacked Transformer encoders until the final encoded sequence *T* is obtained.

During the forward propagation, a mask is generated to ignore padding tokens in the input. The input sequence is first expanded in dimensionality and then transposed. Positional encodings are added to the embedded sequence to preserve temporal structure. The model iteratively processes the sequence through each encoder layer, applying self-attention and feedforward transformations. The use of positional encoding ensures the model can leverage position-related information, while masking ensures correctness when dealing with padded input. Finally, the encoded output is passed through a lightweight convolutional layer and forwarded to the CNN classification head for arrhythmia prediction.

### Optimizer and loss function

To ensure stable training and convergence, we adopt the Adam optimizer with learning rate warm-up and decay strategies. The learning rate increases linearly at the beginning to avoid gradient instability and then decays according to an inverse square root schedule to improve convergence and generalization. A fixed random seed ensures reproducibility. Early stopping is employed to prevent overfitting if validation performance stops improving.

Given the severe class imbalance in arrhythmia data, we use Focal Loss to emphasize learning from minority classes:

5 $$\begin{aligned} \textrm{FL}(p_t) = -\alpha (1 - p_t)^{\gamma } \log (p_t) \end{aligned}$$

The modulating factor $\gamma$ and class weight $\alpha$ help reduce the contribution of easy-to-classify samples and focus on hard examples.

The learning rate *lr* is defined as:

6 $$\begin{aligned} lr = lr_{\text {max}} \times \min \left( \frac{\text {step}}{\text {warmup\_steps}}, \sqrt{\frac{\text {warmup\_steps}}{\text {step}}} \right) \end{aligned}$$

Here, *warmup_steps* is typically 5–10% of the total training steps, and $lr_{\text {max}}$ is tuned via small-scale experiments.

The training pipeline includes forward propagation, loss computation, backpropagation, and parameter updates. After each epoch, the model is evaluated on a validation set, and the best-performing weights are saved. Final predictions are made using a SoftMax layer and evaluated using confusion matrix, accuracy, precision, recall, and F1-score.

### Multimodal feature fusion module

To further improve the discriminative ability and cross-patient robustness of the proposed ECGBert model, we introduce a multimodal feature fusion module that jointly models temporal features extracted by CNN and semantic representations learned by BERT. Although ECG signals are one-dimensional time-series data, they contain rhythm patterns, waveform variations, and sequential dependencies that require multi-level and multi-view representations. A single architecture is often limited in its perception scale, information dimension, and representation granularity, making it difficult to capture both local details and global dependencies simultaneously.

Therefore, we design a cross-modal alignment and gated fusion mechanism between CNN features and BERT embeddings. Compared with simple concatenation or serial architectures, this design offers several advantages: (1) CNN and BERT are trained separately before fusion, reducing gradient interference; (2) the attention mechanism dynamically adjusts the contribution of each modality, improving sensitivity to abnormal rhythms; (3) the fused representation retains both morphological details and global context, enhancing robustness.

**Mathematical Formulation.** Let the CNN output be:

7 $$\begin{aligned} C \in \mathbb {R}^{T \times d_c}, \end{aligned}$$

and the BERT output be:

8 $$\begin{aligned} B \in \mathbb {R}^{T \times d_b}, \end{aligned}$$

where *T* is the number of time steps, and $d_c$ and $d_b$ are the feature dimensions of CNN and BERT, respectively.

First, both features are projected into the same dimension *d*:

9 $$\begin{aligned} C' = C W_c + b_c, \quad B' = B W_b + b_b, \end{aligned}$$

where $$W_c \in \mathbb {R}^{d_c \times d}, \; W_b \in \mathbb {R}^{d_b \times d}$$.

Then, cross-modal attention is applied for alignment:

10 $$\begin{aligned} A = \textrm{softmax}\left( \frac{(C'W_Q)(B'W_K)^\top }{\sqrt{d_k}}\right) , \quad F_{CB} = A(B'W_V), \end{aligned}$$

where $W_Q, W_K, W_V \in \mathbb {R}^{d \times d}$, and $d_k = d/h$ with *h* denoting the number of attention heads.

Finally, a gated fusion mechanism is used:

11 $$\begin{aligned} g_t = \sigma \big (W_g [C'_t ; F_{CB,t}] + b_g \big ), \quad F_t = g_t \odot C'_t + (1-g_t) \odot F_{CB,t}, \end{aligned}$$

where $\sigma$ is the Sigmoid function and $\odot$ denotes element-wise multiplication. The fused feature sequence $F = \{F_1, \dots , F_T\}$ is then fed into the classification layer.

Compared with traditional concatenation or serial fusion, this design has three main advantages: 1) Cross-modal attention enables fine-grained feature alignment; 2) The gating mechanism dynamically balances the contribution of each modality; 3) The fused representation preserves both local waveform details and global temporal dependencies.

### Dataset description

This study employs the publicly available MIT-BIH Arrhythmia Database, jointly established by MIT and Beth Israel Hospital, which is widely used for arrhythmia classification and evaluation. The dataset contains 48 records of 30-minute two-lead ECG signals, sampled at 360 Hz, from 47 subjects aged between 23 and 89. Each record includes annotations for over 110,000 beats, categorized into normal sinus rhythms, atrial premature beats, ventricular premature beats, left/right bundle branch blocks, etc.

The ECG signals were normalized to zero mean and unit variance. A band-pass filter between 0.5 Hz and 50 Hz was applied to remove noise such as baseline drift and power-line interference.To improve model generalization, data augmentation techniques including time stretching and amplitude scaling were used to increase sample diversity.

All heartbeat annotations were reclassified into five standard classes (N, S, V, F, Q), as defined in Table 1.The dataset was divided into 70% for training, 10% for validation, and 20% for testing, as shown in Table 2.

**Table 1:** MIT-BIH annotations mapped to the standard 5-class classification scheme.

Class	Class Name	Symbols	Description
N	Normal	N, L, R, e, j	Normal beats, Left/Right bundle branch block, escape beat, nodal junctional beat, etc.
S	Supraventricular ectopic beat	A, a, J, S	Atrial premature beat, aberrant atrial beat, junctional beat, supraventricular beat, etc.
V	Ventricular ectopic beat	V, E	Premature ventricular contraction, accelerated idioventricular rhythm
F	Fusion beat	F	Fusion of ventricular and normal beat
Q	Unknown/Other beat	f, u	Unclassifiable beat, artifact, unknown beat

**Table 2:** Detailed sample counts of each class in the training and testing subsets of the adopted dataset.

Classes	N	S	V	F	Q
Training	72471	2223	5788	641	6431
Testing	8894	246	700	80	802

### Model parameters

In this section, we evaluate the effectiveness and rationality of our proposed model by comparing it with different baseline models trained on the same dataset.The detailed hyperparameter settings are summarized in this section to ensure reproducibility. The hyperparameter settings of our model are summarized in Table 3.

**Table 3:** The parameter design of the model.

Parameter	Description
num_inputs=1	Input dimension at each time step (usually single-channel for ECG)
num_channels=[1,1,1]	Number of output channels in each TCN layer (affects feature extraction capacity)
kernel_size=5	Kernel size of the TCN (determines the temporal window size)
dropout1=0.3	Dropout rate in the TCN module (prevents overfitting)
d_feature	Length of the input signal
d_model	Embedding dimension in the Transformer (key hyperparameter)
d_inner	Intermediate dimension of the Feed-Forward Network (FFN)
n_layers	Number of Transformer layers
d_layers	Number of TCN layers (possibly customized)
n_head	Number of attention heads in the multi-head attention
d_k, d_v=64	Dimensions of keys and values in the attention mechanism
dropout	Overall dropout rate in the Transformer
class_num	Number of output classes (i.e., types of heart rhythms)

## Results

### Performance metrics

To evaluate the performance of our model, we selected five commonly used classification metrics: accuracy, precision, recall, F1 score, and specificity. These metrics were chosen because they collectively offer a comprehensive assessment of model performance, reflecting both correctness and robustness in clinically relevant scenarios.

The first metric, *accuracy*, measures the overall proportion of correctly classified samples and reflects the global classification performance. It is defined as:

12 $$\begin{aligned} \text {Accuracy} = \frac{TP + TN}{TP + TN + FP + FN} \end{aligned}$$

Where *TP* (true positive) denotes the number of correctly predicted positive samples, *TN* (true negative) refers to correctly predicted negative samples, *FP* (false positive) represents the negative samples incorrectly predicted as positive, and *FN* (false negative) indicates positive samples incorrectly predicted as negative.

*Recall* evaluates the proportion of actual positive samples that are correctly identified:

13 $$\begin{aligned} \text {Recall} = \frac{TP}{TP + FN} \end{aligned}$$

This metric reflects the model’s sensitivity, which is crucial in clinical diagnosis to minimize missed cases.

*Precision* is defined as:

14 $$\begin{aligned} \text {Precision} = \frac{TP}{TP + FP} \end{aligned}$$

It measures the proportion of predicted positive samples that are indeed positive, reflecting the reliability of positive predictions.

*Specificity* quantifies the model’s ability to correctly identify negative cases and is calculated as:

15 $$\begin{aligned} \text {Specificity} = \frac{TN}{TN + FP} \end{aligned}$$

This metric is important to reduce false alarms, which can lead to unnecessary anxiety or treatment.

*F1-score*, the harmonic mean of precision and recall, is calculated as:

16 $$\begin{aligned} \text {F1} = 2 \times \frac{\text {Precision} \times \text {Recall}}{\text {Precision} + \text {Recall}} \end{aligned}$$

It provides a balanced evaluation of the model’s ability to correctly identify and reliably predict positive cases, especially in imbalanced data scenarios.

### Performance of our model

In our experiments, we trained the proposed CNN-BERT model on the MIT-BIH Arrhythmia Dataset to perform classification across five types of heartbeat rhythms. The model demonstrated strong classification performance overall, indicating good generalization ability and high accuracy.

As shown in Table 4, the model performed particularly well on the normal (N) and unknown (Q) classes. The classification accuracy for these two categories reached 99.56% and 99.81%, respectively, while the F1-scores were 99.66% and 99.79%, indicating near-perfect recognition. This outstanding performance is largely attributed to the clear and stable waveform characteristics of the normal class, which is also supported by many samples, allowing the model to fully learn its patterns.

The unknown class often corresponds to artifacts or undefined waveforms with highly irregular features, making it easier for the model to distinguish it from other classes. The model also achieved high precision in identifying supraventricular ectopic beats (S class) and ventricular ectopic beats (V class), indicating strong capability in detecting abnormal heartbeats. For the fusion beats (F class), the model also delivered promising results, with an F1-score of 97.83%. Despite the relatively small number of F-class samples, the distinct waveform features enabled the model to maintain effective recognition.

**Table 4:** The detailed results of CNN-BERT for each class on the MIT-BIH dataset.

Per-class performance	N	S	F	V	Q
Accuracy/%	99.56	93.42	98.69	83.95	99.81
Specificity/%	97.86	99.85	99.91	99.87	99.13
Precision/%	99.56	93.41	98.79	83.95	98.87
Recall/%	99.75	92.28	97.01	85.01	99.42
F1-score/%	99.66	92.84	97.83	84.47	99.79

However, the model showed relatively weaker performance on S class and V class. For the S class, the F1 score was 92. 84% and the recall was 92.28%, indicating that the model had some difficulty correctly identifying these samples. This limitation can be attributed to the small number of S-class samples in the training set and the similarity of their waveform features to those of normal beats, which increases the likelihood of misclassification.

The classification performance was lowest for the V class, with an accuracy of 83.95% and an F1-score of 84.47%. Several factors contribute to this challenge, including the limited sample size, high variability in waveform morphology, and partial overlap with other rhythm types. These issues make it difficult for the model to learn stable and discriminative features for this class. Moreover, V-class arrhythmias often exhibit complex, irregular patterns in clinical scenarios, which are hard to capture using convolution-based features with fixed receptive fields.

Although the CNN-BERT model achieved promising results overall, its relatively weak performance on V class deserves further discussion. We argue that this limitation may be partially attributed to the model architecture itself. CNN modules are effective at extracting local waveform features but rely on fixed receptive fields, which may be insufficient to capture the highly irregular and diverse morphologies of V beats. Meanwhile, the BERT encoder excels at modeling sequential dependencies but may overemphasize global context while underrepresenting subtle morphological variations specific to V beats.

In contrast, ECGTransform incorporates a pure Transformer-based encoder with flexible attention mechanisms across the entire sequence, which may provide greater adaptability to the variability of V beats, explaining its superior performance in this class. Future work could therefore explore enhanced fusion mechanisms that allocate higher attention weights to morphology-sensitive channels or even design a dedicated sub-network tailored to V-beat recognition. Additionally, incorporating domain knowledge (e.g., clinical priors on V-beat waveform morphology) and specialized augmentation strategies may further improve the model’s robustness for this challenging class.

In summary, the CNN-BERT model demonstrated strong performance in the five-class arrhythmia classification task, especially in identifying N, Q, and F beats, where it achieved near-perfect accuracy. The model’s limited performance on the V class suggests that future work could benefit from applying data augmentation to expand the sample size or introducing class-weighted loss functions to improve sensitivity toward minority classes.

The proposed 1D-CNN-BERT architecture offers an effective technical approach for intelligent ECG analysis and assisted diagnosis, showing promising potential for clinical application.

## Discussion

### Performance analysis and comparison

#### Extended experiments and generalization in the nine-class task

To further evaluate the robustness and generalization ability of the ECGBert model in more complex arrhythmia classification scenarios, we extended the original five-class task to a nine-class classification task using the MIT-BIH Arrhythmia Dataset. This setting involves finer-grained heartbeat types, which more closely reflect the complex electrophysiological patterns encountered in real-world clinical practice. It also imposes greater demands on the model’s sequence modeling capability and discriminative precision.

In the nine-class experiment, we maintained the same model architecture and training strategy and evaluated each heartbeat type using standard metrics including accuracy, precision, recall, specificity, and F1-score. As shown in Table 5, the ECGBert model still achieved excellent overall performance under the increased task complexity, reaching an overall accuracy of 99.12% and an F1-score of 95.36%. These results demonstrate the strong scalability and generalization capacity of the model when applied to more demanding classification tasks.

**Table 5:** Performance of the 9-class classification model.

Class	Accuracy/%	Specificity/%	Precision/%	Recall/%	F1 score/%
C1	99.33	98.46	99.33	99.71	99.52
C2	99.51	99.96	99.50	99.75	99.63
C3	99.78	99.98	99.78	99.40	99.59
C4	94.93	99.89	94.93	90.75	92.79
C5	97.27	99.81	97.27	98.19	97.73
C6	92.59	99.98	92.59	84.75	88.50
C7	95.12	99.97	95.12	73.58	82.98
C8	98.89	98.99	98.89	94.68	96.74
C9	99.61	99.97	99.62	99.39	99.50

Specifically, the model maintains high accuracy and stability across most classes. For example, in classes C1 to C3, the F1 scores all exceed 99.5%, indicating that the model can accurately capture their stable and high-frequency waveform features. For classes C4, C6, and C7, which are prone to confusion or have fewer samples, the model also demonstrates robust performance. Although there is a decline in recall and F1 scores–particularly for class C7, with a recall of 73.58% and an F1 score of 82.98%–the overall performance remains satisfactory.

Compared to the five-class classification results, the ECGBert model shows only a slight decrease in average F1 score when the task difficulty increases significantly, dropping from 95.72% in the five-class task to 95.36% in the nine-class task. This reduction is within 0.4 percentage points, indicating strong generalization ability.

Overall, ECGBert continues to deliver excellent performance on the nine-class task, confirming its robustness and generalizability when handling high-dimensional and complex arrhythmia data. These results suggest that the proposed model has good transferability and potential for application in clinical automatic recognition and assisted diagnosis systems for multiple arrhythmia types.

#### Ablation study

We conducted ablation experiments to validate the effectiveness of our proposed architecture. The vanilla BERT model consists of multiple stacked Transformer encoder layers, whereas our ECGBert integrates a one-dimensional convolutional layer to extract local temporal features, followed by a fully connected layer to map the concatenated representations to the final classification output.

Table 6 reports the performance comparison between the CNN-BERT hybrid model and the pure BERT model. It can be observed that ECGBert achieves consistent improvements in accuracy, precision, and F1-score, with the F1-score increasing from 95.274% to 95.904%. This demonstrates that the hybrid model achieves more balanced classification across different classes, particularly enhancing recognition for minority categories. Moreover, CNN-BERT also achieves higher specificity, indicating superior capability in identifying negative samples and thereby reducing the risk of misdiagnosis. These findings confirm the effectiveness of combining CNN and BERT for arrhythmia classification, especially for multi-class, long-sequence ECG signals where both local morphology and global dependencies must be captured.

**Fig. 2:**
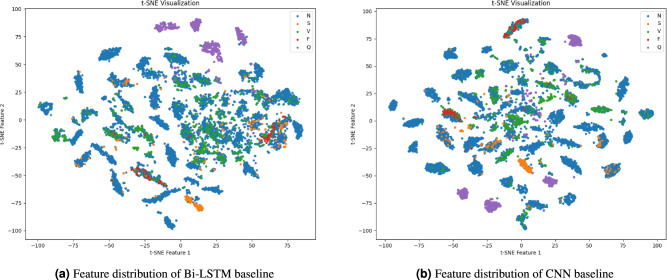
t-SNE visualization of feature representations from different baseline models.

To further investigate the limitations of simple baseline models, we performed t-SNE visualization on the latent feature representations learned by Bi-LSTM and CNN baselines (Figure 2aand Figure 2b). The Bi-LSTM baseline yields highly scattered and overlapping feature clusters, with severe confusion among the N, S, and V classes. The CNN baseline exhibits relatively clearer clustering, particularly for N and Q classes, but substantial overlap persists for S and V classes. These observations suggest that Bi-LSTM emphasizes temporal dependency while CNN focuses on local morphology, yet neither model alone is sufficient to achieve robust inter-class separability. By contrast, our hybrid CNN-BERT model benefits from the complementary strengths of both approaches, producing more compact and discriminative feature clusters, which aligns with its superior quantitative performance shown in Table 6.

Table 6 shows the performance comparison between the two models.

**Table 6:** Ablation study performance comparison.

Model	Accuracy/%	F1-score/%	Specificity/%	Precision/%	Recall/%
CNN-BERT	99.281	95.904	99.500	95.524	95.685
BERT	99.263	95.274	99.459	95.563	94.986

#### Comparison with other models

To comprehensively evaluate the performance of the proposed 1D-CNN-BERT model in ECG classification tasks, we compared it with several previously published models. The results demonstrate that our model outperforms both individual models and existing hybrid models, particularly in the classification of less represented classes. Detailed results are shown in Table 7.

**Table 7:** Per-class recognition performance comparison with other models.

Methods	Metric	N	S	F	V	Q
CNN+Bi-LSTM^30^	Precision	99.60	82.80	87.50	94.57	98.54
	Recall	99.01	92.70	83.33	99.10	99.75
	F1-score	99.30	87.40	85.36	96.78	99.14
ECGTransform^17^	Precision	99.36	91.67	91.30	95.74	99.30
	Recall	99.46	86.91	82.68	97.65	99.06
	F1-score	99.41	89.22	86.78	96.69	99.18
MSH-GCN^25^	Precision	100.00	99.28	100.00	99.72	99.75
	Recall	99.98	99.28	100.00	100.00	99.75
	F1-score	99.88	91.76	100.00	98.38	97.14
CNN-BERT (ours)	Precision	99.56	93.41	98.79	83.95	98.87
	Recall	99.75	92.28	97.01	85.01	99.42
	F1-score	99.66	92.84	97.83	84.47	99.79

For S class, CNN-BERT achieves the highest F1-score of 92.84%, outperforming CNN+Bi-LSTM and ECGTransform. This result indicates that CNN-BERT is more effective in capturing the subtle features of S beats, likely due to the combination of CNN’s local feature extraction and BERT’s global contextual modeling capabilities.

In the F class, CNN-BERT performs particularly well, with an F1-score of 97.83%, significantly higher than CNN+Bi-LSTM (83.54%) and ECGTransform (86.78%). This indicates that CNN-BERT can effectively learn the complex characteristics of fusion beats, whereas conventional models may have limitations in this regard.

However, in the classification of V class, ECGTransform achieves the best performance with an F1-score of 96.69%, followed by CNN+Bi-LSTM (95.34%), while CNN-BERT performs relatively poorly at 84.47%. This may be due to insufficient feature learning for V beats or potential overfitting, indicating that further optimization is needed for this class.

For Q class, all models achieve near-perfect performance, with F1-scores exceeding 99%. CNN-BERT ranks first with a score of 99.79%, suggesting that this task is relatively simple or that the dataset is well-annotated, allowing the models to easily learn its patterns.

In summary, CNN-BERT demonstrates superior performance in most categories (N, S, F, Q), especially in the F class classification. However, its performance on V class is less satisfactory. ECGTransform performs best for class V but shows weaker results in other categories. CNN+Bi-LSTM provides balanced performance across classes but lacks a clear advantage.

Our model architecture also exhibits notable advantages. The 1D-CNN model alone has limited capacity to capture global context, restricting its performance. Although 2D-CNN introduces spatial convolution and enhances local feature representation, its generalization ability remains limited. The AE + Transformer model has strong representation learning capabilities, but its performance is less stable, often affected by class imbalance or convergence issues.

Our model achieves the highest classification accuracy, indicating its effectiveness in recognizing both majority and minority classes. The model not only maintains high precision but also ensures comprehensive coverage of positive samples, reflecting strong generalization ability. Moreover, the loss decreases rapidly in early training and stabilizes after a few epochs, indicating a smooth optimization process without significant overfitting.

### Conclusion

In this study, we proposed ECGBert, a hybrid deep learning model that integrates 1D-CNN and BERT for multi-class arrhythmia recognition. By combining local temporal feature extraction with global contextual modeling, ECGBert enables more accurate and comprehensive ECG analysis.

Experimental results on the MIT-BIH Arrhythmia Database demonstrate that ECGBert consistently outperforms both traditional and hybrid baselines across key evaluation metrics, highlighting its superior accuracy, robustness, and potential for clinical application. The incorporation of attention-based visualization further strengthens its interpretability and clinical reliability, while model optimization strategies suggest promising avenues for deployment in resource-constrained environments.

Nevertheless, despite its strong performance, ECGBert still exhibits limited transparency in its decision-making process, reflecting the inherent ”black-box” nature of deep learning models. To address this issue, future research will explore the integration of explainable AI (XAI) techniques–such as Grad-CAM, SHAP, and Integrated Gradients–to identify the ECG segments that most strongly influence classification outcomes. Comparing these findings with established electrocardiographic features and prior studies will help validate the model’s predictive rationale and enhance its trustworthiness in clinical practice.

Further directions include lightweight model design, cross-database generalization, multimodal physiological signal integration, and the development of privacy-preserving learning frameworks. Taken together, these efforts position ECGBert as a promising step toward practical, intelligent ECG analysis and reliable clinical decision support.

## Data Availability

The datasets analysed in this study are publicly available in the PhysioNet repository at https://www.physionet.org/content/mitdb/1.0.0/. All data processing scripts and trained model files are available from the corresponding author on reasonable request.
